# Measuring what matters to rare disease patients – reflections on the work by the IRDiRC taskforce on patient-centered outcome measures

**DOI:** 10.1186/s13023-017-0718-x

**Published:** 2017-11-02

**Authors:** Thomas Morel, Stefan J. Cano

**Affiliations:** 1 KU Leuven, Herestraat 49, 3000 Leuven, Belgium; 2 Modus Outcomes, Letchworth Garden City, UK

**Keywords:** Patient-centered outcome measures, Rare diseases, Patient-focused drug development (PFDD), Clinical outcome assessments, Patient-reported outcomes, Patient-relevant outcomes, Mixed methods research, Patient centricity, Rasch measurement theory

## Abstract

Our ability to evaluate outcomes which genuinely reflect patients’ unmet needs, hopes and concerns is of pivotal importance. However, much current clinical research and practice falls short of this objective by selecting outcome measures which do not capture patient value to the fullest. In this Opinion, we discuss Patient-Centered Outcomes Measures (PCOMs), which have the potential to systematically incorporate patient perspectives to measure those outcomes that matter most to patients. We argue for greater multi-stakeholder collaboration to develop PCOMs, with rare disease patients and families at the center. Beyond advancing the science of patient input, PCOMs are powerful tools to translate care or observed treatment benefit into an ‘interpretable’ measure of patient benefit, and thereby help demonstrate clinical effectiveness. We propose mixed methods psychometric research as the best route to deliver fit-for-purpose PCOMs in rare diseases, as this methodology brings together qualitative and quantitative research methods in tandem with the explicit aim to efficiently utilise data from small samples. And, whether one opts to develop a brand-new PCOM or to select or adapt an existing outcome measure for use in a rare disease, the anchors remain the same: patients, their daily experience of the rare disease, their preferences, core concepts and values. Ultimately, existing value frameworks, registries, and outcomes-based contracts largely fall short of consistently measuring the full range of outcomes that matter to patients. We argue that greater use of PCOMs in rare diseases would enable a fast track to Patient-Centered Care.

**Electronic supplementary material:**

The online version of this article (10.1186/s13023-017-0718-x) contains supplementary material, which is available to authorized users.

## Background

Rare disease patients are increasingly confronted with a multi-faceted paradox.

First, despite growing acceptance that patients have the clearest view of the health outcomes that matter, the success (or failure) of the majority of rare disease drug development programmes rests on surrogate outcomes (e.g. laboratory measures, organ size) that may not reflect treatment benefits that patients value [1]. Has the rare disease voice been lost in translation?

Second, whilst patients’ plea for new treatments was duly heard and resulted in worldwide efforts to accelerate and intensify rare disease research (as attested by the increase in orphan designations granted by regulatory agencies [2–4]), the regulatory approval and the critically important reimbursement of new treatments for rare diseases are increasingly difficult to obtain. This is due, in part, to the lack of demonstration of improvement in meaningful health outcomes for patients.

The difficult choice of which outcomes to measure, the acceptance of surrogate endpoints, and the question of what represents a meaningful treatment benefit for patients have led to heated debates among regulatory agencies. Drug reviews of new orphan drugs aimed at idiopathic pulmonary fibrosis or Duchenne muscular dystrophy, for example, and the dispute over the relevance of the forced vital capacity and 6-min walk test as study endpoints to predict treatment benefit, are now turning into textbook cases [5–7]. Likewise, health technology assessment (HTA) reviews of new rare disease treatments commonly criticise the clinical effectiveness of orphan drugs due to: a lack of relevant clinical outcomes; uncertainty of what constitutes a minimal clinically important difference; or a lack of validated outcome measures. Statements across HTA reports such as: ‘t*he evidence did not support the achievement of outcomes known to be clinically relevant to patients*’; ‘*the use of [surrogate endpoint] is debatable*’; or ‘*there is a lack of correlation with clinical outcomes that may be more relevant*’ are not infrequent. A recent analysis showed that 38% of negative reimbursement recommendations of drugs for rare diseases in Canada (2004–2015) resulted from a lack of demonstrated clinical effectiveness [8]. And the U.S. Institute for Clinical and Economic Review (ICER) value framework also acknowledges that ‘*all too often what matters most to patients is poorly captured in the available clinical trial data*’ [9].

Third, while encouraging overall clinical trial success rates for rare disease treatments are being reported [10, 11], recurrent late-stage drug development programme failures (e.g. trials investigating novel treatments for Huntington’s disease, myasthenia gravis, systemic lupus or sarcoidosis) have fueled frustration with traditional outcome measures. Core to these failures is the inability to demonstrate statistical significance and meaningful benefit. Failed therapies or flawed outcome measurement?

Today, this lack of consensus about the most important outcomes to study is now contributing to delays or denials of patient access to new treatment options. In the context of severe, devastating, progressive and fatal diseases where fewer than 5% have an approved treatment, this is all very frustrating and, most importantly, raises the opportunity to do something about it.

It was against this background that the International Rare Diseases Research Consortium (IRDiRC) – an initiative launched in 2011 by the European Commission and the U.S. National Institutes of Health to foster international research collaboration and investment in the field of rare diseases – decided in June 2015 to set up a bespoke Task Force on Patient-Centered Outcome Measures (PCOMs). The conclusions from the IRDiRC PCOMs Task Force, published in 2016, provide an excellent overview of the field and highlight that developing patient-centered outcome measures for rare diseases is a ‘*necessity’* [12]. The present Opinion builds on the work by the IRDiRC Task Force and aims to further insist on the critical importance of PCOMs in rare disease research. First, we unpack some of the terminology around PCOMs and their value to healthcare stakeholders, and then expand some of the underlying challenges to outcome measurement in rare diseases. Second, we discuss and illustrate through numerous case studies the various routes to PCOMs in rare disease, with mixed methods research as the main driving force. Finally, we make proposals to build the momentum towards PCOMs in future.

## What are patient-centered outcome measures and what is their value to healthcare stakeholders?

‘*It is clear you have to start with an understanding of the impact of the disease on the people who have it, and what they value most in terms of alleviation before you set up a measurement and go forward with truly patient-focused drug development’* [13]. This sentence by Dr. Janet Woodcock, Director of the Center for Drug Evaluation and Research (CDER) at the U.S. Food and Drug Administration (FDA), captures the central elements of patient-centered outcome measurement.

Whilst PCOMs are not a new idea, their uptake in the research community has been slow and laborious. The recent push to advance the science of patient input and to further incorporate patient perspectives into drug development [14–17], however, has now created a new momentum for patient-centered endpoints that goes beyond long-standing established (or legacy) instruments. The FDA has been instrumental to promote the cause of PCOMs through its ‘Roadmap to patient-focused outcome measurement in clinical trials’ [18]. They are likely to earn greater attention now with the twenty-first Century Cures Act and the forthcoming Prescription Drug User Fee Act (PDUFA) VI in the U.S. [19–22].

What makes PCOMs unique? First, they ‘directly’ quantify the impact of a disease and treatment on health outcomes that matter to patients. Second**,** they are instruments developed with the central concepts of interest (i.e. how patients survive, feel or function [18]) in the foreground, with patient input being sine qua non. PCOMs include but are not limited to self-report instruments: they embrace all forms of clinical outcome assessments (COAs), namely ‘patient-reported outcome’ (PRO), clinician-reported (ClinRO), observer-reported (ObsRO) and performance outcome (PerfO) measures [23]. Third, PCOMs should always be ‘*fit-for-purpose’*. In plain language, they should measure the right outcomes (that resonate with patients’ daily experience of the disease, preferences, expectations and values) in the right patients (i.e. across a continuum of disease severity and manifestations).

**Fig. 1:**
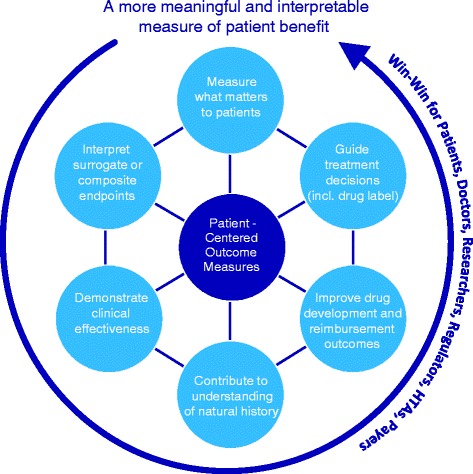
PCOMs bring value across all healthcare stakeholders. Evaluating value from the perspective of the patient can bring substantial benefit for all healthcare stakeholders. Because they are grounded in what matters most to patients, PCOMs help translate care and/or observed treatment effect into an ‘interpretable’ measure of patient benefit. By doing so, PCOMs bring value to all healthcare stakeholders involved. PCOMs may be used for several purposes, such as: efficacy endpoints in clinical trials, outcomes measures in registries, guides to treatment choices for daily care, or tools to monitor care delivery

At present, appropriate and fit-for purpose PCOMs do not exist for most rare diseases, and their use has been largely omitted across the medical and research community. However, if adequately developed, PCOMs have the potential to ‘*speak’* to patients and address their chief complaints. By either directly measuring patients’ clinical function or complementing the use of surrogates [Table 1], PCOMs ultimately offer the opportunity of a more meaningful and interpretable measure of patient benefit – thereby reducing uncertainty over treatment/care effectiveness. Moreover, their use is not limited to clinical studies investigating new drugs and extends to real-life clinical practice (including disease registries) to improve our understanding of the natural course of disease and guide treatment choices (notably through clinical guidelines and regulatory drug labeling). To fully embrace PCOMs (and the necessary investment in time and efforts that come with it), we believe, offers the prospects of a win-win scenario across all healthcare actors (i.e. patients, regulators, researchers, drug developers, HTA agencies, payors, and prescribers). Figure 1 summarises how PCOMs bring value.

**Table 1:** PCOMs bring meaning to surrogate outcomes: the example of ruxolitinib for myelofibrosis [78–80]

*Disease context:* Myelofibrosisis is a rare disease of the bone marrow that disrupts the body’s normal production of blood cells. Sometimes the spleen or the liver takes over some of the blood production; these organs then enlarge which causes abdominal discomfort and pain. Typical symptoms also include feeling of fullness, night sweats and itching. Some patients with myelofibrosis develop leukaemia.
*Research context & question:* Phase 3 study assessing efficacy of ruxolitinib for myelofibrosis. Does shrinking a patient’s spleen lead to a patient-meaningful benefit?
Why add a PCOM? Spleen volume, as such, is a surrogate endpoint that ‘may’ predict treatment benefit, but is not in itself a direct measure of treatment benefit. Thus, as ruxolitinib was being developed, its sponsor chose – after sustained interactions with the U.S. FDA – to supplement the Phase 3 study primary endpoint on the reduction in spleen size with a newly-developed disease-specific patient-reported outcome (PRO) questionnaire (MSAF).
*Result:* Using a direct measure of treatment benefit from treated patients proved to be an effective complement to the primary surrogate endpoint to allow for fast regulatory approval and the avoidance of the requirement for additional post-marketing confirmatory trials. Its impact extended to reimbursement and HTA outcomes, where the improvement in disease-related symptoms were considered to be very important (for example in Germany and Canada), as very aligned with patients’ experience and values. This subsequently allowed patients affected with myelofibrosis to gain access to ruxolitinib as a new treatment option.

## Can we really measure what matters and how?

### Challenges to outcome measurement in rare diseases

Heterogeneity and variability are the two hallmarks of rare diseases. Even within defined conditions, health outcomes (i.e. symptoms and signs of the disease and its overall effect on patient function) can be very different across individuals and these can change as the condition progresses. This is often confounded by the difficulty in distinguishing between the symptoms associated with the rare condition and those due to comorbidities. The main challenge rests in the difficulty to clearly determine and agree on what outcome ought to be measured. This is all the more true as there may not be any discrete outcome that is measurable across the whole patient population.

Take the example of Alström syndrome, a rare complex genetic disorder, associated with a wide variety of symptoms affecting multiple organ systems of the body. Generally characterized by a progressive loss of vision and hearing, it may also involve obesity in childhood, insulin resistance, type 2 diabetes mellitus, dilated cardiomyopathy (i.e. weakening and expansion of the heart) and slowly progressive kidney dysfunction. Additional symptoms including pulmonary, hepatic, renal, and endocrine dysfunction can also occur. This challenge to identify the right outcome is magnified by the small size and geographical spread of the patient population (e.g. only some 1200 individuals affected by Alström syndrome have been identified worldwide to date) and also because half of the individuals affected by rare diseases are children (which raises the question of the reliability of self-reports by young children and the relevance of proxy reports vs. observer reports) [24–26].

Moreover, rarity and poorly understood natural history can sometimes lead to misconceptions about what affects patients at different stages of disease. Thus, clinical information about the disease reported in medical literature may be misleading. And, at times, the outcomes considered critical by clinicians are not the same as the priorities of patients. In chronic, debilitating diseases such as most rare diseases, disease stabilisation ‘*is*’ improvement and may thus be considered as a meaningful outcome to patients [27, 28]. Another hurdle to accurate outcome measurement relates to a phenomenon known as ‘response shift’, which in this case would refer to situations where rare disease patients adapt to their impairment leading to a ‘new normal’; their self-reported health status becomes ‘fine’ [29].

### Patients as partners to understand disease burden

To overcome these challenges it is important to invest in a careful description of the clinical manifestations, disease course, clinical outcomes and – importantly – of the disease impact on patients’ daily life and of the patients’ chief complaints and expectations from future therapies. A comprehensive understanding of patient disease burden is key to later support the assessment and selection of the outcome measures that are most relevant to patients. Because a rare disease affects every aspect of their daily life, patients and their caregivers become experts of the rare condition and of the important outcomes of diseases that need to be addressed. It is thus critically important to partner with and listen to them [30] [Table 2].

**Table 2:** Rare disease patients are the best experts of their conditions [81–84]

*Disease context:* Idiopathic pulmonary fibrosis (IPF) is a chronic and ultimately fatal disease characterized by a progressive decline in lung function. The term pulmonary fibrosis means scarring of lung tissue and is the cause of worsening dyspnoea (shortness of breath).
*US FDA’s commitment to gain the patients’ perspective:* In September 2014, the U.S. FDA held a public meeting to hear perspectives from people living with idiopathic pulmonary fibrosis, its impact on their daily life, and currently available therapies. FDA conducted the meeting as part of the agency’s Patient-Focused Drug Development initiative, an FDA commitment under PDUFA V to more systematically gather patients’ perspectives on their condition and available therapies to treat their condition. At this meeting, patients clearly described the major issues associated with uncontrollable, prolonged episodes of coughing such as: shortness of breath, physical fatigue or overall malaise, and the overall impact on work and home life, including stigma.
*Discordances:* While patients with IPF identified cough as a central symptom during an investigation about core outcome parameters, it did not come out of the Delphi panel of 254 medical experts.
*Result:* It was recognised that the traditional physiological measures measured in clinical trials, such as forced vital capacity (i.e. the amount of air which can be forcibly exhaled from the lungs after taking the deepest breath possible) do not fully capture the potential benefits of a treatment that would matter to individuals affected by IPF. Although cough and fatigue are great concerns in IPF patients, traditional outcome measures have omitted to capture them adequately.

Every route and data source should be explored to overcome the limitations of our current minimal knowledge on rare diseases. Qualitative research is a vital first step to map out the patient and caregiver experience of living with a rare disease. It spans a variety of research methods, with the goal of gathering an in-depth understanding of a patient’s situation, focussing on ‘*what’* and ‘*how’* (i.e. the patient experience of daily living with the rare disease), as opposed to ‘*how much*’ (i.e. analysing statistics). All methods should be considered. The methods commonly used in PCOM research include, but are not limited to the following:

- In-depth pre-trial concept elicitation patient interviews to enable extensive exploration of the disease experience, such as the most significant symptoms and overall disease impact on daily life;
- Interviews in a clinical trial setting (e.g. study exit interviews or the so-called ‘subject experience interviews’ that are spread across the duration of an investigational trial) can bring insightful information on how patients define ‘improvement’ and ‘treatment benefit’;
- Focus groups provide a platform for patients to interact and to compare their experiences;
- Use of internet and social media;
- Direct observation allows researchers to ‘shadow’ patients while doing day-to-day activities to gain first-hand experience through observation of what it means to have a rare disease. This method can be conducted in conjunction with patient interviews to provide rich data; and
- Audio/written diaries provide rare disease patients with an immediate medium to record their experiences.

Of course, other data stemming from literature reviews, disease burden surveys, preference studies, real-world/registry evidence, or placebo and standard-of-care control arms of past clinical trials [31] provide invaluable complementary information. In particular, literature reviews can inform upon qualitative and quantitative research, concepts, and existing instruments and core outcome sets (the latter of which can then be evaluated to determine whether they fully capture patient experience). Databases such as COMET or COSMIN [32, 33] may serve as a useful starting point to track these existing instruments. And of note, the use of internet and social media (e.g. RareConnect) can be an effective leverage to overcome patients’ geographical spread. For example, analysis of internet discussion groups posted by rare disease patients can provide access to unabridged peer-to-peer discussion, free from researcher intervention. This may also enable us to access hard-to-reach subgroups.

Ultimately, the methods used in each instance will depend on a number of constraints. But the more sources tapped into, the better the chance to provide the breadth and depth of information needed to make qualitative comparisons across patients. This leads to the development of conceptual models, which bring together all patient-based evidence and lay out the relationship between core signs, symptoms, concerns, and disease impacts that matter to all (or most) patients with the condition and the hypothesized treatment benefit [Table 3]. By doing so, we can begin to answer: the extent to which the subjective experience of rare disease can be conceptualised; which experiences vary and how; and which existing instruments adequately represent patients’ experience. However, without sufficient information on the disease it can be problematic to conceptualise treatment benefit. This further emphasises the importance of fully integrating patients as partners to understand disease burden.

**Table 3:** Conceptualising the impact of disease [85]

*Disease context:* Phenylketonuria (PKU) is a genetic disorder characterised by a deficiency of the hepatic enzyme, phenylalanine hydroxylase; left untreated, it can lead to intellectual impairment, deficit in cognitive functions, seizures, behavioural problems and psychiatric symptoms.
*Study & methods:* To develop a new PRO instrument based on a conceptual model of PKU through exploratory interviews with clinical experts, adults and adolescents with PKU, and parents with young children with PKU.
*Result:* The conceptual model of PKU impact included health status (cognitive function, symptoms, monitoring); psychological function; social function; and diet. Potential mediators of disease impact included adherence, coping, social support, and other sociodemographic characteristics. For illustration purposes, the conceptual model is available in Additional file 1.

We believe that rare disease patient organisations can steer or even lead (in partnership with all other health stakeholders, including academia) most of the work to map out the ‘*context of use*’ (e.g. rare disease under consideration, stages of disease, sub-populations, healthcare system) and ‘*concepts of interest*’ (e.g. symptoms, functioning) [18]. There are many compelling examples of such leadership role, such as: the Duchenne Parent Project’s outcome measurement initiative in Duchenne non-ambulant boys [34, 35] [Table 4]; the PROBE project in haemophilia [36]; the Dravet syndrome internet platform [37–39]; the Parent Project Muscular Dystrophy work on benefit/risk preferences [28]; or the surveys on patient perspective and priorities led by SMA Europe and Cure SMA [40]. Such leadership role may also result in more ambitious multi-stakeholder forums. For example, in September 2016, the U.S.-based Myotonic Dystrophy Foundation (MDF) hosted the first official externally-led Patient Focused Drug Development (PFDD) meeting, which had an agenda that followed the format of U.S. FDA’s PFDD meetings and aimed to more systematically gather patients’ perspectives about their condition and available therapies [41].

**Table 4:** Rare disease patient advocates shift the focus [34, 35]

*Disease context:* Duchenne muscular dystrophy (DMD) usually presents itself as muscle weakness at around the age of four in boys, which then rapidly deteriorates. Typically muscle loss occurs first in the upper legs and pelvis followed by those of the upper arms. Many are unable to walk by the age of 12 years.
*Drug trial focus:* Until recently, the focus of drug trials in DMD has been the ambulant stage of the disease. Motor function assessment has been the main focus, with the use of the 6-min walk test and ClinROs, such the North Star Ambulatory Assessment.
*A shift in focus:* Since the average age at loss of ambulation is ca. 10.5 years and median survival is 30 years, most individuals affected by DMD are non-ambulant. Under the leadership of the Netherlands-based advocacy group Duchenne Parent Project, a multidisciplinary and multi-stakeholder group identified the need for novel outcome measures for use across the whole spectrum of DMD patients. They developed the Performance of the Upper Limb module (PUL), a ClinRO designed specifically for DMD.
*Adding the patient voice:* In addition to the PUL, this group recognised the need to develop in parallel a patient-reported outcome measure to complement information on daily living that cannot otherwise be observed in a clinical or research setting and focusing on outcomes that are meaningful to patients. As boys and young men with DMD were interviewed in that context, they confirmed that what mattered to them included: *‘to be able to put their arms on the table*’, ‘*to retain the ability to use a computer keyboard’*, ‘*to brush their teeth’*, ‘*to pour a drink*’ etc. – in other words, their hopes focused on retaining upper body function; not necessarily to see improvements in their ability to walk. An example of such a patient interview (for the Upper Limb PRO) is now available online [86].

## When should we select, adapt or develop new PCOMs?

### From clinical concepts to measurement: The power of mixed methods psychometric research in rare diseases

**Fig. 2:**
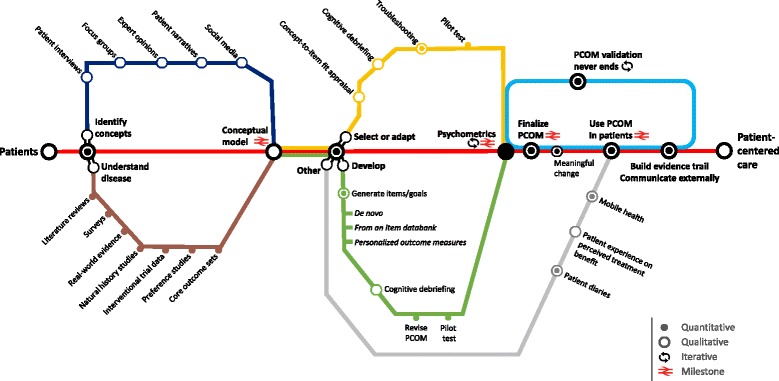
‘On track’ to Patient-Centered Outcome Measurement. To be useful, and provide meaningful information, PCOMs should be grounded in patients and in their daily experience of the rare disease, core concepts, expectations and values. Developing PCOM strategies is an iterative process where qualitative and quantitative patient evidence complement each other to identify those outcomes that matter most to patients. Thus, we always start our journey with the patient (far left of figure) and gain a full understanding of the disease and key concepts (Dark Blue and Brown lines) before proceeding. This may require more than one loop to get correct. Following this, whether one opts for the route to select/adapt an existing outcome measure (Yellow line), or that to develop a novel PCOM (Green line), the anchor remains the same: patients and the conceptual model. Because rare diseases are rare and complex, creativity and pragmatism should prevail. This could include alternate routes to information gathering (Grey line). Ultimately, as we approach our intended destination (Patient-Centered Care), we need to ensure our PCOMs are finalised (Light Blue line), and we are able to begin to build an evidence base for its use

Once the outcomes of interest are identified, an instrument which reflects those outcomes can either be selected, adapted or developed. Whatever the route one may opt for (discussed further below and illustrated in Fig. 2), the parameters remain the same: study samples are going to be small and patient populations are going to be heterogeneous. Therefore, in rare disease research, pragmatism and creativity are required, while maintaining high-standards research practices. This involves moving away from sole dependence on traditional hard psychometric statistics and criteria, and fully integrating what rare disease patients have voiced. As such, the traditional psychometric data-driven approach to PCOM is inherently inappropriate in rare disease because, by definition, there are limited available data to drive the decisions. And thus, there is no inherent constraint on the intelligence we could use in a rare disease context, so we should put the emphasis on the hypotheses and critical thinking to base our decisions, rather than the data. To this end, we believe that mixed methods psychometric research is the best fit in rare diseases. This methodology brings together qualitative and quantitative research methods in tandem with the explicit aim to efficiently utilise data from small samples [42]. The goals of this marriage focus on maximising clinical interpretability, increasing our understanding of the concepts under study, and avoiding potential measurement problems early.

It is fundamental that appropriate conceptual models and definitions are developed in the first instance in the construction of any PCOM. This provides the substantive foundation of any PCOM instrument. The extent to which a conceptual model can then be converted into a list of items (i.e. questions with response options which are assigned numbers), to which the responses of patients or clinicians can be summed to form a total score, requires detailed examination. Of all the measurement properties, ‘*content validity*’ is sine qua non. Any subsequent quantitative analysis (known as psychometrics) is at best limited, and at worst misleading (or meaningless), without this substantive patient-driven clinically-anchored framework. Adequate and appropriate clinically meaningful interpretation of PCOM data rests heavily on this a priori articulation.

Once we have a draft instrument (or are evaluating an existing instrument), three main psychometric approaches can be used to assess the measurement properties of PCOM: Classical Test Theory (CTT), Item Response Theory (IRT), or Rasch Measurement Theory (RMT). Despite having apparent common goals, these approaches differ methodologically, ideologically, and practically. As in any research setting, the psychometric approach needs to be clearly justified after taking into consideration the context of use and concept of interest. All three psychometric approaches can be used in developing PCOMs. But it is widely acknowledged that IRT or RMT include more sophisticated methods than CTT, and that IRT (in the instances where modelling involves multiple parameters) requires large samples to ensure stable estimates. Therefore, RMT provides the most appropriate and scientifically defensible psychometric methods for use in small sample mixed methods research [43–53]. We expand on the central issues supporting Mixed Methods Research and Rasch Measurement Theory in Additional file 2.

Taken together the key properties of RMT speak directly to the core of the utility of rare disease PCOMs: the ability to detect treatment benefit. When sample sizes are necessarily limited, high instrument responsiveness (i.e., the ability to detect all important effects, even if small) is particularly important. This entails appropriately capturing and quantifying those proximal (core) symptoms and their direct impact, which we would expect to be affected by the treatment, to provide the best chance of picking up a treatment benefit signal. Good examples of the use of RMT in rare disease include the improvement of Hammersmith Functional Motor Scale (HFMS) in spinal muscular atrophy [54, 55], the de novo development of the upper limb PCOMs for DMD [34, 35], and strategies to measure clinical change using the North Star Ambulatory Assessment in DMD receiving different corticosteroid regimens [56].

### The routes to PCOMs in rare diseases

Few disease-specific PCOMs are available for use in rare disease. Many commonly used traditional instruments do not relate specifically enough to the disease containing a mix of conceptually different items, some of which are irrelevant or non-applicable. This introduces ‘noise’ rather than ‘signals of patient benefit’ when being used to evaluate a health intervention. That being said, it is practically impossible to develop different specific outcome measures for every rare disease. Therefore, consideration of recycling existing instruments from one context of use to another is worth exploring. Of particular relevance would be considering concept-specific instruments, which may be applicable across a ‘family of rare diseases’ (e.g., autoimmune diseases that share chronic fatigue as a chief complaint, or neuromuscular diseases associated with loss of ambulation) [57].

The choice of any existing instrument should be driven first by careful consideration of item content and the extent to which the specific concept of interest is well-represented in the context of the rare disease target population being studied. Selecting instruments based on their frequency of use in an area, their name (i.e. what they purport to measure), or else based on literature reviews focussing on comparative psychometric properties alone, can be misleading. ‘*Off-the-shelf*’ instruments may not be appropriate, specific or well targeted. Content validity in the selected context trumps all. Ultimately, always go back to the source: patients (and caregivers in the instance of a paediatric rare disease or patients unable to report), and your other anchor: the conceptual model. Here again, the use of mixed methods research can be a powerful strategy to corroborate the appropriateness and range of content, item-to-concept fit, item wording and response option structure of a candidate questionnaire. There are three main potential outcomes at the end of this process: the instrument is deemed fit for purpose in the specific context of use; the instrument is broadly acceptable but requires adaptation [Table 5]; a new instrument with better targeted items and more relevant response options is required.

**Table 5:** Rare disease patients shed light on blind spots and assist in instrument troubleshooting [87–89]

*Disease context:* Amyotrophic lateral sclerosis (ALS) is a neurological disease that attacks the motor neurons, the cells that the brain uses to keep muscles moving. Over the course of three to five years, people with ALS progressively lose the ability to move their fingers and toes, their arms and legs. Then they lose the ability to speak, to turn their head, and to swallow food. When the diaphragm and chest muscles give out, they can no longer breathe and die.
*A legacy instrument:* The Amyotrophic Lateral Sclerosis Functional Rating Scale – Revised (ALFRS-R) is an established rating scale for measuring the global function of patients with ALS.
*Gaps identified and troubleshooting:* When Cathy (a research psychologist affected by advanced ALS) came to complete the ALSFRS-R she was frustrated that despite her ability to participate in family life and write poetry (with the aid of assistive technology, such as an eye tracking machine and a computer to communicate), the scale reflected her as ‘a zero’. When answering the questionnaire: - ‘*Compared to the time before you had symptoms of ALS [...] have you noticed any changes in your speech?*’ She could no longer speak. Zero point. - ‘*Have there been any changes in your ability to swallow?’* She hadn’t swallowed in years. Zero point. - ‘Has your ability to walk changed?’ She could not walk or move her legs. Zero. etc.
*Resolution:* Though a valuable rating instrument, ALFRS-R was deemed not fit-for-purpose in advanced stages of disease. In response, Cathy reached out to the online community PatientsLikeMe to develop new items with input from over 300 ALS patients. Three new items were selected, relating to: the ability to show emotional expression in the face, the ability to use fingers to manipulate devices, and ability to get around inside the home. Subsequent research using Rasch analysis confirmed that a refinement of ALFRS-R was required. The ALSFRS Extension is now used in ALS research.

The development of a new PCOM (either disease- or concept-specific) may thus be warranted where no established measure has been identified to meaningfully capture the patient experience with a rare disease or where not all of the identified patient concepts can be measured through their use. For instance, while established measures of cognition can be used across most rare inborn errors of metabolism, measures of behaviour may require complementary disease-specific scales such as the Sanfilippo Behavior Rating Scale [58]. As illustrated in Fig. 2, several routes are open to PCOM development and include: the development of a brand-new (de novo) PCOM through the design of new items/domains/response options [Table 6]; the use of item databanks to develop a PCOM; and novel PCOM strategies such as personalised outcome measures [Table 7].

**Table 6:** Developing a PCOM de novo: an example of best practice – the Performance of the Upper Limb (PUL) module for DMD [35]

*Objective:* To develop a new measure suitable to cover all the aspects of upper limb function – a concept valued across the whole spectrum of DMD patients (i.e. from younger ambulant to older weaker adults who may only have limited finger movements).
An iterative and multi-stakeholder process: Development of the PUL involved several steps: [1] A systematic review was performed to identify existing measures assessing upper extremity functional aspects used in DMD. Only four ClinROs were found to have been previously used in DMD; [2] An exploratory study was performed to assess the suitability of the existing scales across 61 DMD patients aged 11–30 years. The study identified shortcomings related to posture, pattern of weakness and contractures requiring compensatory strategies; [3] A conceptual model reflecting the progression of weakness and natural history of functional decline in DMD was hypothesized during a multi-stakeholder workshop. Functional tasks were subdivided into three main levels reflecting disease progression from proximal to distal and different stages of the disease: shoulder dimension, elbow dimension, and wrist and finger dimension; [4] An initial set of items was determined based on expert opinion, input from patients and families. Items were refined, added, or eliminated based on feedback; [5] An iterative consultative process with patients, families as well as experts ensured that items in PUL were clinically meaningful and relevant to DMD. Patients and families identified gaps in the proposed assessment; [6] A preliminary pro forma was developed and piloted in 86 patients across seven international sites in Europe and the USA; [7] Rasch analysis was used to create a scale and to review item fit to the underlying construct. A revised version of the PUL including 22 items and a manual were developed and agreed by all the participants. The PUL continues to be reviewed.
*Impact:* A multi-stakeholder collaboration, where patients with DMD and their families had a prominent role, was key to the successful development of the PUL. Modern psychometric methods were used to create a scale with robust internal reliability and validity.

**Table 7:** The aspiration of personalized outcome measurement in rare diseases [90–96]

*Moving beyond the standard:* Many PCOMs, such as ClinROs or PROs, typically include a standard set of items (or tasks) each rated on a standard set of response options, regardless of the relevance of specific items to each individual patient. When rare disease patients are in very different stages of their disease or when a rare disease is ultra-rare and affects a handful of individuals worldwide, these types of instrument may not have sufficient discriminatory capacity to detect change in clinically meaningful dimensions that are important to patients. In other words, a health outcome or an improvement that is relevant or resonates with one patient, may not with another. Two alternatives currently stand out: Goal Attainment Scaling and Computer Adaptive Testing.
*Goal Attainment Scaling (GAS):* GAS allows patients and their treating professionals to work together to identify individual treatment goals that have the greatest relevance. A key feature of GAS is the ‘a priori’ establishment of criteria for ‘successful’ outcomes, which are agreed with the patient and family before a health intervention starts so that everyone has a realistic expectation of what is likely to be achieved and agrees that this would be worth striving for. An example of GAS for use in haemophilia (named GOAL-Hem) covers four broad categories: managing haemophilia (e.g. being able to administer factor), haemophilia complications (e.g. bleeds, pain, joint problems), impact on activities, and impact on emotions and relationships. The applicability of each goal area is determined for different age groups (i.e. adults, adolescents, children). For instance, a common goal for paediatric patients (aged <15) is to become competent and responsible for self-infusion of factor concentrate. This goal area can be selected, current baseline ability assessed and quantifiable degrees of improvement described (a priori) to define potential outcomes.
*Computer Adaptive Testing (CAT):* Whilst CAT has been used most notably in educational testing, the approach has more recently been applied to health outcomes, such as the Patient Reported Outcomes Measurement Information System (PROMIS®) measures and the European Organisation for Research and Treatment of Cancer CAT (EORTC CAT). In CAT, the computer administering the ‘test’ selects questions or ‘items’ from an item bank based on a patient’s response to previously answered questions. Although patients receive different questions based on their individualized responses, scores are standardized and can be compared using a common scale. The goal of CAT is to improve measurement precision for each individual for the specific domain of interest (e.g. physical functioning, depression) being measured using the least number of items.
*Prospects:* GAS and CAT are promising methodologies. But, nonetheless, in relation to PCOM, these are still in their infancy. Whilst GAS has the potential for greater relevance sensitivity over standard measures, the appropriateness of comparing scores between patients has not been proven yet, and is in fact a real challenge. Alternatively, CAT provides a common frame of reference for direct measurement comparability between patients, but items are selected by the computer algorithms with no recourse to patient preferences. One promising initiative developed for visually impaired patients may provide a bridge between GAS and CAT. As such, the Activity Inventory (AI) is an adaptive visual function CAT that consists of 459 tasks grouped into 50 goals. Visually impaired patients rate the importance of each goal, allowing for a CAT to deliver an individually tailored set of items specific to patients.

The relevance of a PCOM relies on its ability to accurately capture the impact of a treatment and communicate those results to patients and other stakeholders. Of particular importance is the degree to which an observed change in a PCOM can be interpreted as clinically meaningful to patients and indicates a treatment benefit. What does a 2-point change on a 0- to 10- point scale mean? Is a 2-point change from 10 to 8 saying the same as a change from 4 to 2? Here again, patients can help researchers define ‘*meaningful change*’. In a rare disease setting where patients are few, emerging novel methods such as the qualitative exit-interview method whereby patients who recently completed a clinical trial are interviewed to provide insight into how observed changes in the measure compare to their own perception of treatment benefit are worth further investigation [59].

Finally, there may be situations where it is unrealistic to aim to accurately measure – with a metric – the impact of a treatment in patients. Such situations include ultra rare diseases that affect a handful of individuals only. Since flexibility and creativity need to prevail in rare disease research, other ‘out-of-the-box’ routes to outcome measurement may be worth a go (illustrated as the Grey line of Fig. 2). Among them is the idea to implement a series of consecutive patient interviews throughout a clinical study to explore with patients the evolution of their treatment experience and perception of treatment benefit. To which extent such qualitative insights may then be converted into quantitative data is a matter of controversy. Patient diaries, a record of the patient perspective of their day-to-day experiences and thoughts relating to their health and medical condition, are another option [60].

## Roadmap towards greater PCOM use in rare diseases

Patient-centered outcome measures (PCOMs) are core to ‘patient-based evidence’ [61] and to the realisation of ‘patient-centered care’ in rare diseases. They highlight the need to systematically include patients in the process of identifying meaningful treatment outcomes that resonate with their experience, preferences, expectations and values [27]. We believe that research and use of PCOMs in the future should be guided by the five principles of: Collaboration, Alignment, Integration, Innovation and Communication.

### Collaboration

PCOM research is a time-consuming and resource-intensive process. Developing PCOMs for a rare disease should be considered as *a non-competitive activity where expertise and resources are pooled*. Inherent to rare diseases is the reality of a ‘rare disease community’, where for many rare conditions a network of patients, patient groups, health professionals, researchers and drug developers work together to promote scientific knowledge to win the fight against the rare condition. This collaborative spirit should be extended to PCOM research. Research and development efforts to ‘crack’ a rare disease never happen out of the blue: they result in the slow accumulation of scientific knowledge that gradually crystallise into clusters of drug development programmes. We suggest that, as clusters of ‘drugable’ targets/pathways emerge and are identified, stakeholders of the targeted rare condition should be encouraged (for instance through ‘Community Advisory Boards’ set up by patient advocacy organisations, as recently proposed by Eurordis [62]) to join forces in early development stages to fund research on what outcomes ought to be measured. Since half of drug developers are small-and-medium-sized companies that don’t necessarily have PCOM expertise, such a collaborative model would boost creative PCOM thinking and result in sharing costs. It would also prevent multiple sponsors developing instruments for the same purpose, which creates inefficiencies. An example of multi-stakeholder collaboration, including patients as leading strategic partners, comes from the haemophilia community. Recent success in gene therapy raises the question whether outcomes that have been used for past clinical trials are suitable for future evaluations of potentially curative technologies. Working in a pre-competitive environment, the CoreHEM project [63–65] seeks to address this question through a multi-stakeholder consensus process, with the goal of agreeing on a standard approach to consistent collection and reporting of relevant and well-specified outcomes, with an emphasis on outcomes deemed most important by the haemophilia community. The project includes a balanced mix of patients, clinicians, haemophilia researchers, US and international payers and health technology assessment groups, governmental entities, and pharmaceutical companies that are currently developing gene therapies for haemophilia.

### Alignment

Since in rare diseases overall patient outcome is the integration of impacts on different domains that the traditional single primary endpoint design is ill-suited to capture well [30], we consider that investigational research should move towards the use of multiple clinical endpoints to ascertain patient benefit. From that end, PCOMs have a central role to play. As clinical development plans are being designed, we believe that the case of patient-relevant outcomes and endpoints should be *discussed proactively with/by regulatory agencies, HTAs and payers*, for instance in the context of joint scientific advice meetings or a qualification procedure [39], so that optimal evidence generation plans are designed and agreed on. As Facey et al. have pointed out ‘*there is a need to gain international agreement on the evidentiary requirements for clinical effectiveness assessments of rare diseases that is accepted by all stakeholders*’ [66]. Of note, there are a number of ongoing initiatives globally (e.g., ICHOM, COMET [67–70]) to develop and agree on standardized collections of outcomes – known as core outcome sets (COS) – to be measured and reported, as a minimum, in all research for a specific clinical area. Their overall aim is to facilitate comparative effectiveness research and evidence synthesis. The Advisory Panel on Rare Disease at the Patient-Centered Outcomes Research Institute (PCORI), for instance, has expressed an interest in developing COS for rare diseases, with a particular focus on paediatric populations [71]. We argue that *patients should be heavily involved in the COS development process*, so that patient evidence guides the adoption of genuine patient-centered COS.

### Integration

Value frameworks and other operational mechanisms to ascertain the value and evidentiary uncertainty of new treatments for rare diseases (such as managed entry agreements, MEAs) are on the rise [72, 73]. However, existing value frameworks largely fall short of consistently measuring outcomes that matter to patients [74]. In order to achieve the overall aim of patient-centered care, we believe that *value assessments must be informed by criteria that matter to patients*. Thus, we encourage the *greater integration of PCOMs into endpoints across both outcomes-based (performance) agreements and patient registries* more broadly. Many registries have limited patient involvement in their design, oversight and operations and the information generated may only partly reflect what matters to patients [75] – this can be reversed by a greater use of PCOMs. Likewise, the outcome measures used to guide start/stop criteria across MEAs to allow patients receive reimbursed treatment are often surrogate or generic measures that may only remotely relate to patients’ disease and treatment experience (as recently exemplified by the English MEA on mucopolysaccharidosis type IVa [76]). Incorporating fit-for-purpose instruments that focus on outcomes that truly matter to patients would enhance patients’ acceptance of the burden brought by managed entry agreements on their daily life. In addition, it would also support the legitimacy of ‘stop decisions’ by health authorities to take patients off treatment.

### Innovation

Where patient evidence suggests that novel PCOMs or the adaptation of existing outcome measures to make them more patient-relevant are warranted, regulatory and HTA agencies should be open to the prospect of innovative measures or methodologies (such as individualised outcome measures) to capture patient benefit. As our knowledge on natural history for every single rare disease keeps increasing, we must be ready to challenge the established order and any ‘clinically validated’ endpoint altogether – *rare diseases require a dynamic model of PCOMs*. Of note, we believe that any rare disease PCOM strategy in the context of investigational trials should be mindful of and compatible with novel trial designs to encourage accelerated development of rare disease therapies. Of note, *mobile health technologies* (wearables, wireless medical sensors, apps etc.) can add to PCOM creativity and hold the promise for improving the quality of patient care and clinical outcomes [77]. Wearables that can unobtrusively measure physiological performance (e.g., movement, vital signs, seizures) offer the opportunity for continuous monitoring and may enhance the ability of researchers to understand the effect of new drugs. Remote assessment (in which the evaluator and individual being evaluated are not located in the same physical space) may also offer significant advantages in the field of rare diseases as they can reduce burdensome travel for patients and families and increase patient access to research studies (while reducing overall costs of collecting patient data). Patients should be involved in the design and operationalisation of such devices.

### Communication

For a PCOM virtuous circle to come into play, open communication is key. We are aware of very interesting ongoing PCOM research initiatives in rare diseases – sadly, their outputs are too often under-published, including their foundational patient qualitative works. This is regrettable since *awareness of best practices can only further promote the cause of PCOMs and also help avoid duplication of efforts*. Scientific publications and presentations to both learned audience and a wider public should occur all along as the evidence on a PCOM is building on. Likewise, training materials (for instance in the context of summer schools for patient representatives) should be developed to empower patients and patient advocacy groups steer PCOM research in future.

## Conclusion

Rare disease therapies should be developed to treat patients, not just their disease: our ability to evaluate outcomes that reflect real benefits from the patient perspective is thus of pivotal importance. Current clinical research and practice, however, are not quite there yet. A multi-stakeholder collaboration should emerge, with the rare disease patient community at its center, to promote the development and use of Patient-Centered Outcomes Measures to help achieve Patient-Centered Care across all rare diseases.

## Additional files

Additional file 1: Conceptual model of the impact of phenylketonuria (PKU) and its treatment on patients and their parents. (DOCX 22 kb)

Additional file 2: Mixed Methods Research & Rasch Measurement Theory – ‘*Le mieux est l’ennemi du bien*’. (DOCX 58 kb)

## Abbreviations

ALS: Amyotrophic lateral sclerosis
CAT: Computer adaptive testing
CDER: Center for Drug Evaluation and Research
ClinRO: Clinical-reported outcome
COA: Clinical outcome assessment
COMET: Core Outcome Measures in Effectiveness Trials initiative
COS: Core outcome set
COSMIN: COnsensus-based Standards for the selection of health Measurement Instruments initiative
CTT: Classical Test Theory
DMD: Duchenne muscular dystrophy
FDA: U.S. Food and Drug Administration
GAS: Goal attainment scaling
HTA: Health technology assessment
ICER: U.S. Institute for Clinical and Economic Review
ICHOM: International Consortium for Health Outcomes Measurement
IPF: Idiopathic pulmonary fibrosis
IRDiRC: International Rare Diseases Research Consortium
IRT: Item Response Theory
MEA: Managed entry agreement
ObsRO: Observer-reported outcome
PCOM: Patient-centered outcome measure
PCORI: Patient-Centered Outcomes Research Institute
PDUFA: U.S. Prescription Drug User Fee Act
PerfO: Performance outcome
PFDD: Patient-focused drug development
PKU: Phenylketonuria
PRO: Patient-reported outcome
PUL: Performance of the Upper Limb module
RMT: Rasch Measurement Theory
SEM: Standard error of measurement
