# Supplementary material for: Measuring what matters to rare disease patients – reflections on the work by the IRDiRC taskforce on patient-centered outcome measures
Source: Orphanet J Rare Dis. 2017 Nov 2;12:171. doi: 10.1186/s13023-017-0718-x (PMC5667521; doi:10.1186/s13023-017-0718-x)
Supplement: Supplementary file 1 — Conceptual model of the impact of phenylketonuria (PKU) and its treatment on patients and their parents. (DOCX 22 kb) [file 13023_2017_718_MOESM1_ESM.docx]

Additional file 2

**Mixed Methods Research & Rasch Measurement Theory – ‘*Le mieux est l’ennemi du bien*’**

Mixed methods psychometric research is an umbrella term; one size does not fit all. A multi-disciplinary approach is almost always required. But different studies will require different emphasises on the complementary qualitative and quantitative methodologies, and different types, kinds and degrees of patient involvement. Whatever the context of use, the chosen research methods should be conducted with rigour, careful design, application, and interpretation. As opposed to simply post-hoc amassing previously collected qualitative data together with some clinical study quantitative data.

Mixed methods psychometric research requires an iterative, hypothesis-based, experimental approach to provide preliminary information to help guide scale development or appraisal. This synthesis of methodologies is important, especially in rare disease. This is because qualitative methods alone are unable to inform us about the extent to which concepts are measurable including: how well targeted the items are to the type and range of issues experienced by patients; how well the items work together as a set; and whether the response options are working as intended. Conversely, quantitative methods alone cannot inform us upon which concepts should be, and/or are purported to be, measured.

Regardless of quantitative methodology (i.e., classical test theory [CTT], item response theory [IRT], Rasch Measurement Theory [RMT]), broadly speaking, psychometricians fall into one of two ideological camps: empirical (i.e., those who prioritise the data and statistical modelling criteria) vs. hypothesis-driven (i.e., those who place precedence in content and measurement principles). A hard-line statistical data-driven approach rests heavily upon the strength of the data used. Hence, this kind of approach is unrealistic for rare diseases, where data are scarce. IRT is a paragon of a data-priority-led ideology, especially in the instances where modelling involves multiple parameters. As such, given larger sample size requirements, IRT becomes a less favourable option for rare disease context. This leaves two remaining psychometrics methods: CTT or RMT. It is possible to imagine a way to apply both in an an iterative, hypothesis-based experimental approach.

The apparent simplicity of CTT (e.g., the possibility to use techniques suitable for small samples, such as non-parametric statistics) could make it appear immediately appealing. However, we would proffer that even in a more general context (i.e., beyond rare diseases), the limitations of CTT-based methods outweigh the benefits [1]. This is because: ordered counts are not interval measures (thus, the unit of measurement implied by the data is not equal across the whole range of the continuum); results for scales are sample dependent (thus, the measurement scale is affected by the sample it is supposed to be measuring); results for samples are scale dependent (thus, people’s measurement on a concept is dependent on the scale on which they are measured and the sample within which they are measured); missing data cannot be handled directly (thus, the oft-used replacing missing data with the person-specific mean score raises a number of concerns as these make questionable assumptions); there is a lack of scaling items (thus, CTT-based methods do not provide items values that locate them on the measurement continuum); and the error around an individual person’s score (standard error of measurement; SEM) is a constant value regardless of the person’s location on the measurement continuum.

Quantitative analysis on small samples sizes can lead to large statistical uncertainty (i.e., standard errors and their implied confidence intervals). But, when CTT is used, we are unable to gain useful information about measurement uncertainty. This is because: 1) SEM is a sample dependent statistic; 2) it’s typically large leading to the inference of limited precision; and 3) it assumes a scale has the same precision at the extremes as in the centre of the measurement continuum, which is unlikely at best. This leads to the problem of managing uncertainty, which is intrinsic to the rare disease context.

RMT [2] overcomes the serious limitations of CTT, as it provides detailed information at the item-level, as well as important information about measurement uncertainty. The cornerstones of the Rasch model are statistical sufficiency, parameter separation and invariant comparison. RMT’s benefits include, but are not limited to: the ability to construct linear measurements from ordinal-level data, thereby addressing a major concern of using PCOMs as outcome measures [3]; providing item estimates that are free from the sample distribution and person estimates that are free from the scale distribution, thus allowing for greater flexibility in situations where different samples or conceptually-similar instruments are used [1]; allowing for the use of subsets of items from each scale rather than all items from the scale, without compromising the comparability of measures made, using different sets of items (the foundation for item banking and computerised adaptive testing) [4]; enabling estimates suitable for individual person analyses rather than only for group comparison studies[5]. These properties provide a strong foundation for detailed diagnosis of PCOM item performance and measurement potential. And, despite often voiced concerns, it is a technically simple approach [6].

There are two potential concerns to our proposition of mixed methods research using RMT in a rare disease context. First, the opinion that by invoking RMT we are potentially misusing a “statistical model, based on statistical theory, and relying on statistical assumptions” (page 2690) [7]. Second, the frequently proffered phrase ‘*perfection is the enemy of the good*’, in this instance referring to mixed methods research being too burdensome and not always being required to produce useful PCOMs.

To address the first concern, we recommend qualitative and quantitative research findings are synthesised to provide an evidence base for how well a PCOM performs as an *instrument of measurement* [8-10]. By using RMT, the quantitative foundations of this rests on a measurement model, based on measurement theory, and relying on measurement requirements [11].

In relation to the second concern, we would say that we also subscribe to Voltaire’s famous quote *‘le mieux est l’ennemi du bien’*. But we prefer its alignment with the original Italian as ‘*Il meglio è nemico del bene’* (‘the better is enemy of the good’) by [Orlando Pescetti](https://en.wikipedia.org/w/index.php?title=Orlando_Pescetti&action=edit&redlink=1) [12]. The fuller Voltaire quote sheds further light as it continues: *‘Non qu’on ne puisse augmenter en prudence/ En bonté d’âme, en talents, en science/ Cherchons le mieux sur ces chapitres-là’* (‘Not that we cannot improve in prudence/ Kindness, skills, science/ Let’s seek for the best on these matters’).

Ultimately, qualitative research is necessary but not sufficient, and quantitative research is necessary but not sufficient. We would argue that rare disease patients need and deserve better patient-centred outcome measures. Mixed methods psychometric research provides the best route to deliver fit-for-purpose instruments.

**References**

1. Hobart, J. and S. Cano, Improving the evaluation of therapeutic interventions in multiple sclerosis: the role of new psychometric methods. Health Technol Assess, 2009. 13(12): p. iii, ix-x, 1-177.

2. Andrich, D., Rating scales and Rasch measurement. Expert Rev. Pharmacoeconomics Outcomes Res., 2011. 11(5): p. 571-585.

3. Grimby, G., A. Tennant, and L. Tesio, The use of raw scores from ordinal scales: time to end malpractice? J Rehabil Med, 2012. 44(2): p. 97-98.

4. Choppin, B., An item bank using sample free calibration. Nature, 1968. 219: p. 870-872.

5. Hobart, J.C., S.J. Cano, and A.J. Thompson, Effect sizes can be misleading: is it time to change the way we measure change? J Neurol Neurosurg Psychiatry, 2010. 81(9): p. 1044-8.

6. Wright, B. and M. Stone, Best test design: Rasch measurement. 1979, Chicago: MESA Press.

7. Houts, C., et al., A review of empirical research related to the use of small quantitative samples in clinical outcome scale development. Qual Life Res, 2016. 25: p. 2685–2691.

8. Cano, S., et al., On Trial: the Compatibility of Measurement in the Physical and Social Sciences. Journal of Physics: Conference Series, 2016. 772(2016): p. 012025.

9. Fisher, W. and A. Stenner, Integrating qualitative and quantitative research approaches via the phenomenological method. International Journal of Multiple Research Approaches, 2011. 5(1): p. 89-103.

10. Pendrill, L., Using measurement uncertainty in decision-making & conformity assessment. Metrologia, 2014. 51: p. S206.

11. Cano, S., et al., Patient-Centred Outcome Metrology for Healthcare Decision-Making. Journal of Physics: Conference Series, 2017. (IN PRESS).

12. https://en.wikipedia.org/wiki/Perfect_is_the_enemy_of_good.
