# Supplementary material for: Measuring what matters to rare disease patients – reflections on the work by the IRDiRC taskforce on patient-centered outcome measures
Source: Orphanet J Rare Dis. 2017 Nov 2;12:171. doi: 10.1186/s13023-017-0718-x (PMC5667521; doi:10.1186/s13023-017-0718-x)
Supplement: Supplementary file 2 — Mixed Methods Research & Rasch Measurement Theory – ‘Le mieux est l’ennemi du bien’. (DOCX 58 kb) [file 13023_2017_718_MOESM2_ESM.docx]

Additional file 1

**Conceptual model of the impact of phenylketonuria (PKU) and its treatment on patients and their parents**


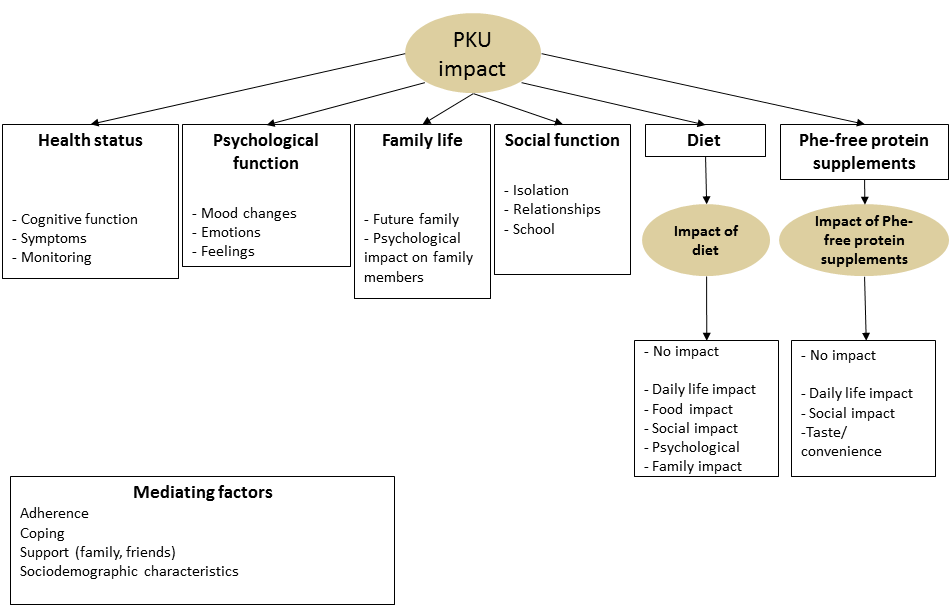


Source: Regnault A, Burlina A, Cunningham A, Bettiol E, Moreau-Stucker F, Benmedjahed K, Bosch AM; Development and psychometric validation of measures to assess the impact of phenylketonuria and its dietary treatment on patients' and parents' quality of life: the phenylketonuria – quality of life (PKU-QOL) questionnaires; Orphanet J Rare Dis. 2015 May 10;10:59.
